# Point of Care Ultrasound in Detection of Brain Hemorrhage and Skull Fracture Following Pediatric Head Trauma; a Diagnostic Accuracy Study

**Published:** 2019-09-24

**Authors:** Maryam Masaeli, Mojtaba Chahardoli, Sepehr Azizi, Babak Shekarchi, Foroogh Sabzghabaei, Nima Shekar Riz Fomani, Mahdi Azarmnia, Mahdis Abedi

**Affiliations:** 1 Emergency Department, Besat Hospital, School of Medicine, AJA University of Medical Sciences, Tehran, Iran.; 2 Emergency Department, Firouzgar Hospital, School of Medicine, Iran University of Medical Sciences, Tehran, Iran.; 3 Department of Radiology, School of Medicine, AJA University of Medical Sciences, Tehran, Iran.; 4 Department of Medicine, Firouzgar Hospital, School of Medicine, Iran University of Medical Sciences, Tehran, Iran.

**Keywords:** Emergency Medicine, Pediatrics, Craniocerebral Trauma, Skull Fractures, Intracranial Hemorrhages, Ultrasonography

## Abstract

**Introduction:**

Head trauma is a common reason for emergency department visits worldwide; many of which involve young children. We sought to determine if head ultrasound (US), as a portable, fast and safe modality, can guide diagnosis and treatment of children in emergency settings.

**Methods:**

In this cross-sectional study, brain computed tomography (CT) scan and emergency head US were performed on head trauma children who were referred to the emergency departments of Firouzgar and Besat Hospitals, Tehran, Iran, from September 2018 to May 2019. The findings of the two modalities were separately evaluated, and used to estimate the diagnostic accuracy of US.

**Results:**

538 patients with the mean age of 5.6 ± 4.9 (0-18) years were studied (54.8% male). Sensitivity and specificity of bedside US in detection of hemorrhage were 85.71% (42.13%-99.64%) and 97.99% (94.23%-99.58%) for children below the age of 2. These measures were 80.00% (51.91%-95.67%) and 97.97% (94.88%-99.44%), respectively, for those between 2 and 6 years old and 46.67% (21.27%-73.41%) and 92.90% (87.66%-96.40%), respectively, for those above the age of 6. Sensitivity and specificity were 92.31% (84.01%-97.12%) and 95.87% (93.62%-97.50%), respectively, in diagnosing skull fractures. Cohen's kappa coefficient varied greatly for different findings, ranging from 0.363 to 0.825, indicating different agreement rates for each.

**Conclusion:**

Based on our findings, emergency US can play a greater role in the initial management of head trauma children, especially as a triage test.

## Introduction

Head trauma is a common reason for emergency department visits worldwide; many of which involve young children. In the civilian population, almost 75% of head trauma is due to falls and motor vehicle accidents (1). Blast-induced Traumatic brain injury (TBI) is also considered a major cause of neuropsychological morbidity during and after wars (2, 3). 

Early presentation of mild TBI is rather nonspecific and may include persistent headaches, vertigo, memory loss and poor concentration (4). The situation needs to be tackled immediately, since any delay in diagnosis and treatment of intracranial injuries can result in rapid deterioration, death, or permanent neurological sequel. The standard imaging test for head trauma patients is non-contrasted computed tomography (CT) scan of brain (5). It is estimated that about one million children undergo unnecessary CT in the USA each year (6). No surprise that various clinical decision rules have been proposed for decreasing its excessive use (7). A number of reasons limit CT application: it is expensive, it is not available in smaller centers, and it employs ionizing radiation; which is especially worrying in children. Furthermore, sedation is very often required for performing the procedure in young children, which poses an extra risk (8).

Point-of-care ultrasonography (US) is increasingly used as a diagnostic tool in emergency departments (9). US is fast, safe, portable, cost-effective and well tolerated even by children (10). But the critical question remains open, as to how promising it is for clinical decision making concerning head trauma. Some evidence suggests that US is accurate for pediatric skull fractures (11, 12). Interestingly enough, there is 20 times more risk of suffering an intracranial injury, for those who are found to have a skull fracture (13). Less research has been conducted on detection of traumatic brain hemorrhages via US. To our knowledge, there are no studies discussing US diagnosis of cranial hemorrhages except for newborns and infants. Although there is still doubt about suitability of US for these areas due to the known attenuating effect of bone on ultrasonic waves (14, 15), the rewards of developing US to a triage tool, particularly for disasters and tactical conditions, are huge. We sought to determine if head US performed by emergency physicians, can guide diagnosis and treatment of children in emergency settings. 

Based on the above mentioned reasons, the present study aimed to evaluate the accuracy of point of care US in detection of brain hemorrhage and skull fractures following pediatric head trauma.

## Methods

***Study design and setting***

In this prospective cross-sectional study, brain CT-scan and head US were performed separately for head trauma children who were referred to emergency departments of Firouzgar and Besat Hospitals, Tehran, Iran, from September 2018 to May 2019 (a 7-month period). The findings of the two modalities along with patients’ demographic data (age, sex), were then collected using a checklist by a trained emergency medicine resident and were used to estimate the diagnostic accuracy of US in detection of brain hemorrhage and skull fractures, by relying on CT-scan as the reference test. 

The study protocol was reviewed and approved by ethics committee of AJA University of Medical Sciences (Ethics code: IR.AJAUMS.REC.1397.118) and researchers adhered to the principles of Helsinki Declaration. There was no interference with routine management of patients, since ultrasounds were performed while patients were awaiting transfer to the radiology department. Before enrollment, informed consent was obtained from the patients or their surrogate decision makers.

***Participants***

Patients younger than 18 years presenting within 24 hours of head trauma were studied. Sources of subject recruitment were emergency departments of Firouzgar and Besat Hospitals, which are among tertiary referral hospitals, in Tehran, Iran. The two centers had common head trauma management protocols. To identify children who were candidates of head CT-scan, the PECARN prediction rule was used (6). PECARN recommends obtaining head CT imaging and observation in the presence of altered mental status, palpable skull fracture, scalp hematoma, loss of consciousness, vomiting, severe headache, not acting normally according to parents, and severe mechanism of injury (6). These patients were enrolled for also having a bedside US evaluation using non-probability sampling. 

Uncooperative patients, patients with GCS≤13 and those with acute hemodynamic instability were excluded. Patients who met the aforementioned criteria were selected consecutively.

***Imaging details***

Point of care US was performed by attending emergency physicians and trained third year emergency medicine residents, and head CT-scans were interpreted by Radiology department. Third year emergency medicine residents were first trained in an emergency US workshop, including a theoretical session as well as hands-on practice, under supervision. The training course met the requirements of the current guideline by the American College of Emergency Physicians (9).

For detection of brain hemorrhages, the children were assigned to three age groups; patients under 2 years old, 2 to 6 years old, and 6 to 18 years old. As the bones continue to ossify, high-impedance difference between bone and soft tissue prohibits visualization of the underlying structures. Accordingly, the 2 years old point was chosen due to the fact that the closure of anterior fontanel is expected to occur by maximum of 18-24 month (16). The ossification further continues so that the Spheno-ethmoidal Synchondrosis is lost at 6-7 year (17). This produces another plausible cut-off since the cranium is 90% of the adult size at this time (18).

US was performed using 2-5 MHZ transducer, phased array, SonoSite M-Turbo machines. The Scalp was first examined for soft-tissue swelling, bruising and other local signs. Cortical irregularities were noted for diagnosing linear and depressed fractures. For detecting blood, echogenicity patterns were probed. Subdural and epidural hematomas commonly present as hypoechoic fluid collections surrounding the brain parenchyma (19). Examinations began using bilateral trans-temporal approach. In infants, anterior and posterior fontanels were also used as fine acoustic windows to the cranial vault. The occipital window was reserved for patients with ruled out cervical spine injury. Then, patients were transferred to obtain their CT scan. On CT, acute extra-axial hemorrhages are usually hyper-attenuating. Hyperacute unclotted blood may be close to water attenuation, while mixed high and low attenuation pattern suggests active bleeding. Skull fractures are also seen in 85% to 95% of cases with epidural hematoma (20). 

The findings of brain CT scan were interpreted by radiologists and the research team later reviewed CT scan reports. At the time of performing the procedures, the US and radiology teams were blind to each other's findings. Individual discussion with surrogate decision makers was used to allay anxiety when recruiting patients. Investigators made all examinations on a single occasion, and no further follow-up was scheduled after the Emergency Department visit.

***Data gathering***

Patients’ baseline characteristics as well as head US and brain CT scan findings were collected for patients using a predesigned checklist by a trained emergency medicine resident.

**Statistical Analysis**

The minimum required sample size considering 17% prevalence of abnormal brain CT scan findings in suspected mild TBI patients (21), 94% estimated sensitivity of US in pilot, 95% confidence interval (α=0.05), and estimation error of 5% (d= 0.05), was calculated as 510 subjects. We finally enrolled 538 cases to avoid errors due to unexpected dropouts. 

Data was transferred to Statistical package for social sciences (SPSS) software version 22. Mean and standard deviation were used to report quantitative data. Qualitative data were presented as frequency and percentage. Cohen's Kappa coefficient was calculated, as a measure of inter-rater agreement between brain CT scan and head US reports. Sensitivity, specificity, positive likelihood ratio, negative likelihood ratio, positive predictive value, and negative predictive value of US in detection of intracranial hemorrhage and skull fracture, were calculated using MedCalc's statistical software, considering CT reports as the reference test. In all cases, p<0.05 was considered significant. Since there were very few missing values, we used casewise deletion to address missing data. 

**Table 1:** Baseline characteristics of studied patients

**Variable**	**Number (%)**
**Gender**	
Male	295 (54.8)
Female	243 (45.1)
**Age (year)**	
<2	156 (28.9)
2-6	212 (39.4)
6-18	170 (31.5)
**Trauma Mechanism**	
Falls	236 (43.8)
Motor vehicle accidents	82 (15.2)
Other	220 (40.8)
**Hypotension**	
No	489 (90.8)
Yes	49 (9.1)
**Loss of Consciousness (GCS < 15)**	
No	478 (88.8)
Yes	60 (11.1)
Headache*	
No	312 (57.9)
Yes	226 (42.0)
**Vomiting**	
No	492 (91.4)
Yes	46 (8.5)
**Seizure **	
No	530 (98.5)
Yes	8 (1.48)

*For ≥2 years’ cases. Hypotension: systolic blood pressure < 90 mmHg. GCS: Glasgow coma scale.

**Table 2:** Agreement between head ultrasonography and brain computed tomography (CT) scan findings of studied patients

**Variables**	**True positive n (%)**		**Cohen’s kappa**	**Strength **
	**Ultrasonography**	**CT scan**		
**Brain hemorrhage**				
<2 years	6 (3.8)	7 (4.4)	0.73 (0.49 - 0.98)	Good
2-6 years	12 (5.6)	15 (7.0)	0.75 (0.58 - 0.93)	Good
6-18 years	7 (4.1)	15 (8.8)	0.36 (0.13 - 0.58)	Fair
**Fracture**				
Skull	72 (13.3)	78 (14.4)	0.82 (0.75 - 0.89)	Very good

**Table 3:** Screening performance characteristics of ultrasonography in detection of brain hemorrhage and skull fracture following head trauma

**Characteristics**	**Brain hemorrhage**			**Skull fracture**
	**<2 years**	**2-6 years**	**6-18 years**	
True positive	6	12	7	72
False negative	1	3	8	6
False positive	3	4	11	19
True negative	146	193	144	441
Sensitivity	85.7 (42.1-99.6)	80.0 (51.9-95.6)	46.6 (21.2-73.4)	92.3 (84.0-97.1)
Specificity	97.9 (94.2-99.5)	97.9 (94.8-99.4)	92.9 (87.6-96.4)	95.8 (93.6-97.5)
PPV	66.6 (38.5-86.4)	75.0 (52.4-89.1)	38.8 (22.4-58.2)	79.1 (70.8-85.5)
NPV	99.3 (95.9-99.8)	98.4 (95.9-99.4)	94.7 (91.8-96.6)	98.6 (97.1-99.3)
PLR	42.5 (13.3-135.8)	39.4 (14.4-107.3)	6.5 (3.0-14.4)	22.3 (14.3-34.8)
NLR	0.15 (0.02- 0.90)	0.20 (0.07-0.56)	0.5 (0.3-0.9)	0.08 (0.04-0.17)

All measures are presented with 95% confidence interval (CI). PLR: Positive likelihood ratio; NLR: Negative likelihood ratio; PPV: Positive predictive value; NPV: Negative predictive value.

## Results

***Baseline characteristics of studied cases***

A total of 538 consecutive head trauma children with the mean age of 5.6 ± 4.9 years (range: 0-18) were studied (54.8% male). Traumas were most commonly due to falls (43.8%). 49 patients were hypotensive (9.1%). A period of loss of consciousness was reported in 60 cases. (11.1%) Table 1 depicts baseline characteristics of the patients. 

***Screening performance characteristics***

The agreement rate between US and brain CT scan in detection of brain hemorrhage and skull fractures is presented in table 2. The lowest and highest agreement between US and brain CT scan reports were regarding detection of hemorrhage in 6 to 18 year-old patients (Kappa: 0.363) and skull fracture (Kappa: 0.825), respectively. 

Screening performance characteristics of head US in detection of brain hemorrhage and skull fractures are summarized in table 3. Sensitivity and specificity of bedside US in detection of hemorrhage below the age of 2 were 85.71% (95%CI: 42.13%-99.64%), 97.99% (95%CI: 94.23%-99.58%), respectively. In children between 2 and 6 years old the results were 80.00% (95%CI: 51.91%-95.67%) and 97.97% (95%CI: 94.88%-99.44%), respectively. Above the age of 6 the sensitivity and specificity were 46.67% (95%CI: 21.27%-73.41%) and 92.90% (95%CI: 87.66%-96.40%), respectively. For diagnosing skull fractures, sensitivity and specificity of US were 92.31% (95%CI: 84.01%-97.12%) and 95.87% (95%CI: 93.62%-97.50%), respectively. 

## Discussion

Based on the findings, the strength of agreement between head US and brain CT scan in diagnosis of skull fractures, and hemorrhages in the age groups of under 2 years, 2 to 6 years and 6 to 18 years can be categorized as very good (k:0.82), good (k:0.73), good (k:0.75) and fair (k:0.36), respectively. 

A very good agreement (Kappa: 0.825) between head US and brain CT scan regarding the absence or presence of skull fracture, is generally consistent with prior studies. For instance, Parri et al. reported 100% sensitivity and 95% specificity of US in diagnosing skull fractures (11). Point-of-care US of Rabiner et al. on 69 patients under the age of 21, also reported 88% sensitivity and 97% specificity in detection of skull fracture (22). In another study, Steiner and colleagues examined 210 children with head trauma. They recommended employing US as a screening test for skull fracture, so the patients with negative US and normal neurological status could only be observed (23). Similarly, US had a higher negative predictive value than positive predictive value in our study.

Mccormick and colleagues assessed the accuracy of US performed by emergency physicians in diagnosis of traumatic intracranial hemorrhage (ICH) in infants. In their study, physicians demonstrated a range of 50% to 100% sensitivity (kappa: 0.4) (24). Our study may be the first to explore the feasibility of emergency US for diagnosing brain hemorrhages in children with head trauma. Independent comprehensive reviews of evidence regarding pediatric emergency medicine point of care US in 2014 and 2016, did not report any evidence on this issue (25, 26). In our study, the strength of agreement between emergency US and CT scan was good for detection of bleeding up to 6 years of age (kappa: 0.737 and 0.756 for 0 to 2 years old and 2 to 6 years old, respectively). However, in older children a dramatic fall was obvious in agreement between US and CT scan (kappa: 0.363). 41.6% (n: 5) of US false negative cases were further diagnosed and localized by CT scan as small frontal bleedings. Under the age of 2, 100% (n: 3) of US false positive cases were misdiagnosed as small temporal ICH, which were further ruled out by brain CT scan. 

## Limitations

One of the limitations of the present study, is that we cannot know how introducing US in the management would affect patients' outcome. Developing head US into a reliable part of clinical decision making requires information on patients' prognosis. 

Our results may also need further confirmation for their generalizability. Although we conducted a multi-center study, both institutions were tertiary large hospitals. Therefore, we recommend that researchers conduct similar studies in primary and secondary care settings of smaller facilities. US results depend on the operators’ training, skill and experience, so accuracy may vary from operator to operator. We did not study inter-observer variability of US findings. 

It is indispensable that the scope of emergency US be clearly defined by further investigators, so that medical associations will be able to issue guidelines on appropriate method of application, evaluation and certification of US in pediatric head trauma.

## Conclusion:

Based on the findings of the present study, there was a very good agreement between head US and brain CT scan findings regarding the absence or presence of skull fracture. The strength of agreement was slightly lower for detection of bleeding in children up to 6 years of age. This can make US a suitable option for initial assessment of head trauma in infants and young children. Yet, significantly lower agreement in those above 6 years old demonstrates the necessity of not overgeneralizing the capabilities of US in older children. Still, it may prove beneficial in disasters. 
